# Ensembles generated from crystal structures of single distant homologues solve challenging molecular-replacement cases in *AMPLE*

**DOI:** 10.1107/S2059798318002310

**Published:** 2018-03-02

**Authors:** Daniel J. Rigden, Jens M. H. Thomas, Felix Simkovic, Adam Simpkin, Martyn D. Winn, Olga Mayans, Ronan M. Keegan

**Affiliations:** a Institute of Integrative Biology, University of Liverpool, Crown Street, Liverpool L69 7ZB, England; b Science and Technology Facilities Council, Daresbury Laboratory, Warrington WA4 4AD, England; c Fachbereich Biologie, Universität Konstanz, 78457 Konstanz, Germany; d Research Complex at Harwell, STFC Rutherford Appleton Laboratory, Didcot OX11 0FA, England

**Keywords:** distant homologues, *CONCOORD*, molecular replacement, search-model ensembles, *AMPLE*

## Abstract

Molecular replacement (MR) is the predominant route to solution of the phase problem in macromolecular crystallography. Although routine in many cases, it becomes more effortful and often impossible when the available experimental structures typically used as search models are only distantly homologous to the target. Nevertheless, with current powerful MR software, relatively small core structures shared between the target and known structure, of 20–40% of the overall structure for example, can succeed as search models where they can be isolated. Manual sculpting of such small structural cores is rarely attempted and is dependent on the crystallographer’s expertise and understanding of the protein family in question. Automated search-model editing has previously been performed on the basis of sequence alignment, in order to eliminate, for example, side chains or loops that are not present in the target, or on the basis of structural features (*e.g.* solvent accessibility) or crystallographic parameters (*e.g.* *B* factors). Here, based on recent work demonstrating a correlation between evolutionary conservation and protein rigidity/packing, novel automated ways to derive edited search models from a given distant homologue over a range of sizes are presented. A variety of structure-based metrics, many readily obtained from online webservers, can be fed to the MR pipeline *AMPLE* to produce search models that succeed with a set of test cases where expertly manually edited comparators, further processed in diverse ways with *MrBUMP*, fail. Further significant performance gains result when the structure-based distance geometry method *CONCOORD* is used to generate ensembles from the distant homologue. To our knowledge, this is the first such approach whereby a single structure is meaningfully transformed into an ensemble for the purposes of MR. Additional cases further demonstrate the advantages of the approach. *CONCOORD* is freely available and computationally inexpensive, so these novel methods offer readily available new routes to solve difficult MR cases.

## Abstract 2

Novel ways to produce search models from distant homologues for molecular replacement are presented.

## 1. Introduction   

Molecular replacement (MR) remains the most popular means to solve the phase problem in macromolecular crystallo­graphy. It requires that a search model be placed, usually through sequential rotational and translational searches, in the asymmetric unit in such a way as to provide helpful phase information and allow the calculation of initial electron-density maps (Rossmann & Blow, 1962 ▸). Search models are still obtained predominantly from experimental structures that are recognisably homologous to the target and so are likely to share some degree of structural similarity with it. Some degree of processing of the characterized homologues is often carried out to remove (portions of) side chains or surface loops which sequence comparison shows to be different, or that are likely to adopt a different conformation in the target. Unconventional MR employs different kinds of search models including ideal secondary-structure elements or other regular motifs (Rodríguez *et al.*, 2012 ▸), recurring tertiary packing arrangements (Sammito *et al.*, 2013 ▸), *ab initio* structure predictions (Bibby *et al.*, 2012 ▸; Keegan *et al.*, 2015 ▸; Simkovic *et al.*, 2016 ▸) and even, for very high resolution cases, single atoms (McCoy *et al.*, 2017 ▸).

Conventional MR using an experimental structure becomes increasingly difficult as the structural differences between it and the unknown target increase (https://www.phenix-online.org/documentation/reference/mr_overview.html; Abergel, 2013 ▸). This structural divergence generally follows sequence divergence and therefore increases with the passage of evolutionary time and the accompanying accumulation of mutations. Thus, cases where the target shares only distant homology with structurally characterized relatives can be highly challenging, and novel approaches to such cases have the potential to significantly extend the reach and ease of MR.

Making the best use of distant homologues for MR can be seen as best identifying the structure that is shared between them and the target, while eliminating the more structurally divergent portions that will only impede structure solution. It was shown by Schwarzenbacher and coworkers that careful preparation of a distant homologue for use in MR, based on a sequence alignment between it and the target, is important for successful placement of the search model (Schwarzenbacher *et al.*, 2004 ▸). Several applications have been developed (Stein, 2008 ▸; Bunkóczi & Read, 2011 ▸; Lebedev *et al.*, 2008 ▸) to perform this task by taking (as input or by creating) a sequence alignment between a target and a homologue, and using this information to truncate the corresponding atomic coordinates for the homologue to produce the MR search model. The main goal of these applications is to identify what is conserved between the homologue and the target and remove the differences. This in turn increases the correlation between the structure-factor amplitudes generated by the search model, once correctly placed by MR in the unit cell of the target, and those of the experimental data. Using advanced alignment methods such as *PSI-BLAST* (Altschul *et al.*, 1997 ▸; Schäffer *et al.*, 2001 ▸) and *FFAS* (Jaroszewski *et al.*, 2005 ▸) to ensure the accuracy of the alignment, in addition to truncating surface-accessible side chains and others with high *B* factors, was found to be crucial to success in cases where the sequence identity of the homologue was below 35% (Bunkóczi & Read, 2011 ▸). Since no single strategy will be suitable in all cases, several automatic pipelines, such as *MrBUMP* (Keegan & Winn, 2007 ▸, 2008 ▸; Keegan *et al.*, 2011 ▸), *BALBES* (Keegan *et al.*, 2011 ▸; Long *et al.*, 2008 ▸), *MRage* (Bunkóczi *et al.*, 2013 ▸) and more recently *MoRDa* (Vagin & Lebedev, 2015 ▸), have been developed. These will find and prepare many search models according to a range of protocols, before trialling them in MR.

Other developments such as *sculpt_ensemble* from *PHENIX* (Adams *et al.*, 2010 ▸) combine the process of truncating several homologues with aligning them to produce an ensemble search model. Ensemble or composite search models can give an additional advantage in the maximum-likelihood scoring approach used by *Phaser* (McCoy, 2004 ▸). Here, the variance in the aligned search models making up the ensemble can guide the weighting of the experimental data and also help in the packing function (McCoy *et al.*, 2007 ▸). As demonstrated by studies using *AMPLE* (Bibby *et al.*, 2012 ▸, 2013 ▸; Keegan *et al.*, 2015 ▸), the truncation of ensemble search models at different structural variance thresholds can help to obtain ensembles representing core regions of the aligned search models which may structurally match the corresponding region in the target. In a comparable approach, but one informed by the experimental data, *ARCIMBOLDO_SHREDDER* (Sammito *et al.*, 2014 ▸) identifies regions of a distantly homologous structure to use or discard by rotation-function scoring of ‘shreds’ systematically obtained by omitting sets of residues.

The maximum-likelihood scoring (Storoni *et al.*, 2004 ▸; McCoy *et al.*, 2005 ▸; Read & McCoy, 2016 ▸) used in *Phaser* (McCoy *et al.*, 2007 ▸) has allowed a greater tolerance of differences between the search model and the target structure than in previously existing MR programs. Errors in the calculated values for the structure factors stemming from both the inaccuracies of the search model and the measurement of the experimental intensities are accounted for by the method, helping to better identify the correct placement of the search model. Recent improvements to *Phaser* (Oeffner *et al.*, 2013 ▸), such as the use of a variance-r.m.s. calculation to better estimate the r.m.s.d. between the search model and the target, have further enhanced its chances of success when a distant homologue is used as a search model. The successful placement of a search model significantly different in its structural conformation from the target can present a problem for refinement. *MR-Rosetta* (Terwilliger *et al.*, 2012 ▸) can assist in such cases by using the *ab initio* modelling functionality of *ROSETTA* (Shortle *et al.*, 1998 ▸; Leaver-Fay *et al.*, 2011 ▸) in combination with *phenix.autobuild* (Terwilliger *et al.*, 2008 ▸) to rebuild search models positioned by *Phaser*.

The identification of conserved structural cores is somewhat more straightforward when several experimental structures of homologues are available. In such a case, the structures can be superimposed using software such as *GESAMT* (Krissinel, 2012 ▸). Regions that are considered to be divergent can then be directly identified and removed. An extension to *CCP*4*mg* (McNicholas *et al.*, 2011 ▸) to visualize superpositions and use a slider to decide how much divergent structure to remove is described elsewhere (Keegan *et al.*, 2018 ▸). The same paper also describes how *MrBUMP* can now carry out graded *AMPLE*-style truncations of a superposition of a selection of user-supplied structures. This allows the user to easily trial ensemble search models derived from a set of distant homologues across a range of sizes. Prediction of the shared structure between the target and a single distantly homologous structure is more challenging. Currently, this might entail the construction of a sequence alignment containing both the homologue and other available sequences, and then mapping that conservation onto the known structure to guide its editing. However, this supposes that a useful amount of sequence information is available, which is not always the case, and would be labour-intensive: the preparation of anything more than a handful of search models would try the patience of the most committed crystallographer.

In this work, we explore automated ways to process a single distant homologue into sets of search models, both as a single trimmed model and as a computationally generated ensemble. The latter can be generated using distance geometry methods applied to the homologous structure. This work was largely prompted by recent bioinformatics work that has demonstrated a good correlation between evolutionary conservation and protein rigidity (Shih *et al.*, 2012 ▸; Yeh *et al.*, 2014 ▸). Tightly packed regions are both more rigid and, since they are less accommodating of sequence change, more evolutionarily conserved. This opens the way for the use of metrics that inform on packing or rigidity as proxies for evolutionary conservation, which may be time-consuming or impossible to calculate directly from sequence alignment and analysis. Such proxy metrics may be as simple to calculate as a weighted contact number or solvent accessibility. Indeed, we demonstrate some success with editing a single structure based on these metrics and solving nontrivial cases. However, we find that an approach based on building a structural ensemble that reflects innate flexibility through being based on geometrical constraints identified in the starting structure is much more effective. This approach, using the software *CONCOORD* (de Groot *et al.*, 1997 ▸), shows promise to facilitate and enable solution by MR in cases of distant homology that would otherwise be difficult or impossible by current methods.

## 2. Methods   

### 2.1. Test-set selection   

A set of seven distant homologues spanning branch 1 of the histidine phosphatase superfamily (Rigden, 2008 ▸) were used to explore the novel MR approaches below. The PDB codes of branch 1 members were retrieved from Pfam (Finn *et al.*, 2016 ▸; entry PF00300), and *CD-HIT* (Fu *et al.*, 2012 ▸) was applied to their sequences in order to obtain a maximally diverse set. Two structures were subsequently added from the exceedingly divergent branch 2 of the superfamily (PF00328). Pairwise sequence identity and C^α^ r.m.s.d. comparisons are shown in Supplementary Table S1. Other characteristics of the targets are shown in Table 1 ▸ along with quantitative structural comparisons of the targets with PDB entry 3c7t made using *GESAMT* (Krissinel, 2012 ▸) and *TM-align* (Zhang & Skolnick, 2005 ▸). Percentage sequence identities between the targets were also measured after alignment with *MAFFT* using the *L-INS-i* accuracy-oriented algorithm (Katoh & Standley, 2013 ▸). The structures of the proteins in the test set were obtained from crystal forms containing 41–63% solvent which diffracted to resolutions ranging from 1.3 to 2.45 Å. Target protein sizes covered an almost threefold size range from 156 residues (PDB entry 1ujb) to 434 residues (PDB entry 1qwo). Two phosphoglycerate mutases sharing around 50% sequence identity were included to assess the impact of resolution on success since one (PDB entry 1e59) was determined at a very high resolution (1.3 Å) while the other (PDB entry 4eo9) was only at moderate resolution (2.45 Å). From this set, the structure of ecdysone phosphate phosphatase (PDB entry 3c7t, 259 residues; Chen *et al.*, 2008 ▸) was randomly chosen as the source of search models with which to attempt the solution of its distant relatives.

### 2.2. Search-model generation   

In this work, three types of search model were used as detailed in the sections below. The first was derived from *CONCOORD* (de Groot *et al.*, 1997 ▸) and the second from a single structure truncated using a variety of per-residue scores considered to potentially reflect rigidity as a proxy for evolutionary conservation, or conservation directly (see also Supplementary Table S2). The third was a set of manually edited crystal structure derivatives.

### 2.3. *CONCOORD*-generated ensembles   

Single structures were converted to structural ensembles with *CONCOORD* (de Groot *et al.*, 1997 ▸) to attempt the solution of distant homologues. *CONCOORD* runs encompass two steps carried out by the programs *dist* and *disco*, respectively. The first program *dist* defines geometric constraints based on the input structure. These constraints comprise firstly the covalent and noncovalent interactions identified and secondly acceptable distance separations between interacting atoms that are required in the structures generated later: stronger interactions are required to satisfy tighter separation criteria. This step can use different van der Waals and bond/angle parameter sets. Here, the default OPLS-UA (Jorgensen *et al.*, 1996 ▸) and *CONCOORD* parameters were used. It also requires the freely available *DSSP* software (Kabsch & Sander, 1983 ▸) for secondary-structure assignment. The *dist* step produces files called dist.dat and dist.pdb that are required for the following step. The second program, *disco*, derives multiple structures from the set of geometric restraints using distance geometry methods. Corrections are applied to initially random coordinates until all restraints are satisfied. Nonconverging runs are discarded and restarted. 500 output structures were generated using default parameters. Prior to running *CONCOORD*, any selenomethione residues present must be reverted to regular methionines and alternative conformations eliminated. Only the latter was required in the case of 3c7t. Sample command lines for *CONCOORD* would be
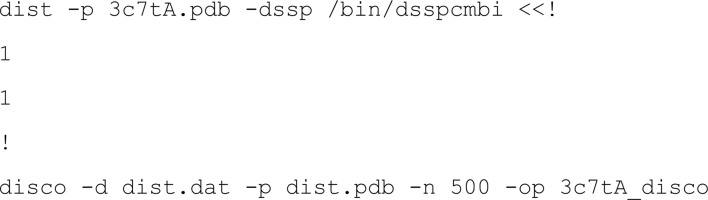


and would produce files 3c7tA_disco1.pdb to 3c7tA_disco500.pdb.

The resulting set of 500 structures were processed with *AMPLE* (Bibby *et al.*, 2012 ▸) in the same way as *ab initio* structure predictions. Briefly, progressive truncations at 5% intervals were carried out based on the per-residue structural variance scores output from *SPICKER* (Zhang & Skolnick, 2004 ▸) clustering. Thus, for a protein of 100 residues, the complete structural ensemble would be taken, along with search models containing superpositions of 95, 90, 85 … 5 residues. Initially, only the largest cluster was used to derive search-model ensembles by truncation and three different side-chain treatments. Two of these were full retention or complete removal leaving polyalanine. The third entailed the retention of only those considered as more reliably predictable by *SCWRL* (Krivov *et al.*, 2009 ▸): this set contains broadly those with fewer well occupied rotamers and hence those that are more likely to be maintained in the same conformation. Ensembles are constructed using three subclustering radii (Bibby *et al.*, 2012 ▸). This procedure leads to a total of 180 search-model ensembles (20 truncation steps × three subclustering radii × three side-chain treatments) being generated per cluster. For cases in which this failed to solve a structure, search models derived from clusters 2 and 3 were additionally trialled, summing 540 search models in all.

### 2.4. Single-structure editing using rigidity and packing metrics   

The single structure of *Bombyx mori* ecdysone phosphate phosphatase (PDB entry 3c7t) was processed according to a file containing per-residue scores (described below) using a newly introduced *AMPLE* mode. This new mode is run as follows.
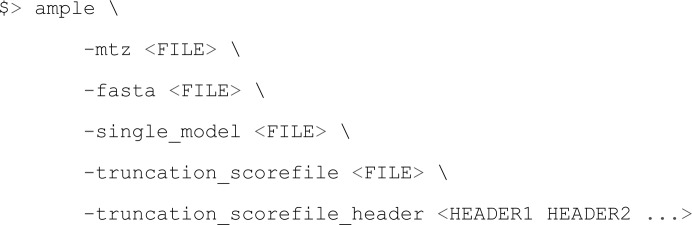


The PDB input (file defined by the -single_model flag) is truncated over the same size intervals and with the same side-chain treatments as above, but the residues that are removed first are those with the highest values in the accompanying file of per-residue scores (as specified by the -truncation_scoref ile flag). This file contains at least two columns, the first being residue numbers and the second values to guide the progressive elimination of residues. The first line of this file, the header, contains the column titles as specified by the -truncation_scoref ile_header flag. Further columns may be present in the scorefile, each with a column title in the header, representing further values by which model editing will occur. These trigger independent processing of the input PDB file according to the specified column values. The results of processing according to values in columns 2–*n* form a single pool of search models which are then trialled in the usual fashion by *MrBUMP* as part of the overall *AMPLE* scheme.

Files of scores, which can also be seen as profiles, were obtained by the following methods (see Supplementary Table S2 for details) and trialled individually for successful MR against the panel of targets. The first set of per-residue scores reflecting flexibility were calculated using the anisotropic network model webserver *ANM* (Eyal *et al.*, 2015 ▸), the coarse-grained dynamics method *CABS-flex* (Jamroz *et al.*, 2013 ▸), *CONCOORD* (de Groot *et al.*, 1997 ▸) as above, and the normal-mode server *WEBnm@* (Tiwari *et al.*, 2014 ▸). More specifically, the *B* factors predicted by *ANM* were used. From *CABS-flex*, the per-residue trajectory fluctuations were taken. The structural variances per residue derived from analysis of *CONCOORD* structures with *THESEUS* (Theobald & Wuttke, 2006 ▸) were used. From *WEBnm@*, residue-fluctuation scores were again taken.

A per-residue score based on packing was measured directly using the weighted contact number (WCN) calculated using the *(PS)* ^2^ server (Huang *et al.*, 2015 ▸). Sequence-conservation scores were calculated using the *ConSurf* server (Ashkenazy *et al.*, 2016 ▸), where five iterations of *CS-BLAST* (Angermüller *et al.*, 2012 ▸) were used with the number of homologues considered in the calculation set to the maximum of 500. The SMRF metric (Jeong & Kim, 2016 ▸) reflecting structure-based covariance was calculated using *SMRF* v.0.4 with default parameters. *ROSETTA* (Leaver-Fay *et al.*, 2011 ▸) refinement using the *relax* protocol with the -relax:fast flag was also tested since less well packed regions would be expected to show more structural variability after repeated refinements (Park *et al.*, 2015 ▸). As with *CONCOORD*, per-residue structural variance scores for the resulting 100 relaxed structures were calculated with *THESEUS* (Theobald & Wuttke, 2006 ▸). Residue-averaged crystallographic *B* factors were also trialled for editing of 3c7t, as were solvent-accessible surface area (ASA) values in Å^2^ calculated for a single 3c7t subunit using the *PISA* server (Krissinel & Henrick, 2007 ▸). Finally, the *ResQ* server (Yang *et al.*, 2016 ▸) was also used to derive scores of predicted residue quality and predicted *B* factor. These are calculated using support-vector regression based on a set of templates identified in the PDB by threading and structural alignment.

### 2.5. Manually edited search models   

For comparison with these metric-driven search models, the PDB structure 3c7t was subjected to manual processing based on examination of the structure. Four different derivatives resulted (Supplementary Fig. S1) containing 88, 95, 138 and 159 residues, in which loop regions had progressively been removed more or less strictly applying the same editing approach as generally adopted in MR.

### 2.6. Molecular replacement   

Sets of search-model ensembles and the automatically processed versions of PDB entry 3c7t were trialled in *AMPLE* 1.01 (or 1.2.0 for the ASA search models only) within *CCP*4 distributions 7.02–7.04 (Winn *et al.*, 2011 ▸), with success being defined as a *Phaser*-2.6.1 (Read & McCoy, 2016 ▸; McCoy *et al.*, 2007 ▸) placement that led to main-chain tracing using *SHELXE* 2016 (Thorn & Sheldrick, 2013 ▸) giving a correlation coefficient (CC) of >25% with an average chain length (ACL) of >10. The default *AMPLE*-estimated r.m.s.d. error of 0.1 Å was used. All of these solutions could be refined to *R*_free_ < 0.45, typically using *ARP*/*wARP* (Langer *et al.*, 2008 ▸) or *Buccaneer* (Cowtan, 2006 ▸) as built into the *AMPLE* pipeline but also, in the hardest cases, with further rounds of *SHELXE* and/or manual iteration between *ARP*/*wARP* and *Buccaneer* for manual rebuilding. The four manually generated search models were processed by *MrBUMP* (Keegan & Winn, 2008 ▸) using default parameters, *i.e.* testing each individually with side chains either left, removed entirely or processed in three different ways (*CHAINSAW*, *MOLREP* and *Sculptor* modes). The *MrBUMP* run additionally attempted structure solution with *MOLREP* (Vagin & Teplyakov, 2010 ▸), although this resulted in no successes. The criteria defining successful MR were the same as above. Software versions for the *MrBUMP* processing of manually derived 3c7t-based search models were *CCP*4 6.5.001/7.0.017, *Phaser*-2.5.6/2.6.1, *MOLREP* 11.2.08/11.4.06 and *SHELXE* 2014/4 or *SHELXE* 2016/3.

### 2.7. Additional examples   

Through collaboration, the *CONCOORD* approach was tried on two further cases: 2,4′-dihydroxyacetophenone dioxygenase from *Alcaligenes* sp. (deposited in the PDB as entry 4p9g; Keegan *et al.*, 2014 ▸) and an unpublished complex between two *Salmonella enterica* proteins (PDB entry 5hxg; B. Li, Y. Yue, Z. Yuan, F. Zhang, Y. Liu, P. Li, N. Song, Z, Li, L. Gu & L. Qin, unpublished work). *CCP*4 distributions 7.0.35 and 7.0.32 were used, respectively. *Phaser* 2.7.17 and *SHELXE* 2016/3 were used in both cases.

## 3. Results and discussion   

### 3.1. Conventional MR from a combination of manual and automatic editing   

Our set of targets represent genuinely challenging distant homology cases (Table 1 ▸): only one shared over 20% sequence identity with the crystal structure with PDB code 3c7t, which was used to derived the search models, in a *MAFFT* (Katoh & Standley, 2013 ▸) multiple-sequence alignment. We therefore produced a set of four manually edited derivatives of 3c7t to obtain an authentic impression of the performance of conventional MR, although it should, of course, be acknowledged that there is an element of subjectivity in the model preparation. The four edits were prepared with increasing degrees of truncation to attempt to capture the most likely features of 3c7t to be conserved in each of the three targets. *MrBUMP* derived additional models from those input using its set of protocols for model preparation. These conveniently and automatically replicate the approaches, such as stripping off all side chains, that a crystallographer would be likely to attempt. This resulted in an additional four search models for each of the inputs, three ‘mixed models’ based on sequence alignment between the target and the input model (*CHAINSAW*, *MOLREP* and *Sculptor*), and a polyalanine version of the input model.

Of the entire set of 20 (four original manual edits and 16 derived) models, only two resulted in a successful solution for just one of the target cases: PDB entry 2qni. These were a polyalanine derivative of the second most truncated model (which gave a *Phaser* TFZ of 7.1 and LLG of 45 and a *SHELXE* CC of 51.13%) and a *MOLREP*-mode search model from the third most truncated model (TFZ = 6.6, LLG = 25; *SHELXE* CC = 51.32%). Both successes used *Phaser* to position the model and *SHELXE* for density modification and main-chain tracing. Notably, none of the original manually processed versions of 3c7t provided a solution; some additional modification by *MrBUMP* was required, indicating how sensitive MR is, in difficult cases, to search-model preparation.

A comparison of structural similarity between the four manual edits and the targets (Supplementary Table S1) offers an explanation for the isolated success of 2qni. It shows that although 2qni has low sequence identity with 3c7t, structural similarity measured by the *Q*-score from *GESAMT* (Krissinel, 2012 ▸) shows it to be the most similar. The *Q*-score is a measure of structural similarity that takes into account the number of matched residues, the r.m.s.d. of the match and the numbers of residues in both matched proteins. For example, with respect to the 138-residue derivative, 110 residues superimpose on PDB entry 2qni with a C^α^ r.m.s.d. of 1.63 Å. Superpositions for other targets, involving 106–123 residues, yield r.m.s.d. values above 1.9 Å. This exemplifies, as is well known, that structural similarity (which is not known in advance) rather than sequence similarity (which is known in advance) is key to success by MR, and that the latter can be an imperfect proxy for the former.

### 3.2. *CONCOORD* ensembles   

The results of attempting to solve a range of distant homologues using *CONCOORD*-derived structures based on PDB entry 3c7t are shown in Table 2 ▸. It is immediately evident that *CONCOORD*-derived search-model ensembles significantly outperform the manual edits. Six of eight targets could be solved compared with the single success of the manual edits. Some successes were obtained using search models derived from the largest cluster of input structures. At the time of the work, *AMPLE*’s default operation was to heavily sample the largest cluster derived from the input models. [This mode is currently available by specifying -classic_mode True at the command line or *via* a GUI.] Three of the six targets in branch 1 of the histidine phosphatase superfamily were solved in this way, but neither of the targets in branch 2 were. Where success was not achieved, clusters 2 and 3 were sampled, increasing the number of search models tested from 180 to 540. In this way, two additional targets from branch 1, 4eo9 and 1ebb, were also solved, along with 1qwo from branch 2.

The solution of PDB entry 4eo9, the 2.45 Å resolution structure of phosphoglycerate mutase from *Mycobacterium leprae* (Baugh *et al.*, 2015 ▸), was somewhat surprising for two reasons. The first was that its resolution is outside the range that is generally considered to be tractable for phase modification and main-chain tracing by *SHELXE*, yet it produced *SHELXE* statistics of a correlation coefficient of 39 and an average chain length of 38. These statistics are indicative of successful solution and, indeed, it could be automatically refined to an *R*_free_ of 0.2303 after five building cycles in *ARP*/*wARP*. Secondly, the homologous enzyme from *E. coli*, determined to 1.3 Å resolution as PDB entry 1e59, could not be solved. However, using a higher 0.5 Å r.m.s.d. error estimate with *Phaser*, as opposed to the default 0.1 Å estimate, did solve this structure (data not shown) and points the way to further refinement of this method. The comparative ease with which 2qni was solved (Table 2 ▸) was explored by determining r.m.s.d. values for the superposition of differently sized successful search models and the target itself (Supplementary Fig. S2). The largest search models can be largely superimposed to within a C^α^ r.m.s.d. of 2.0 Å, while the loss of scattering matter in more truncated versions seems to be offset by low r.m.s.d. values of around 0.75 Å, ensuring success over a broad size range.

**Figure 1:**
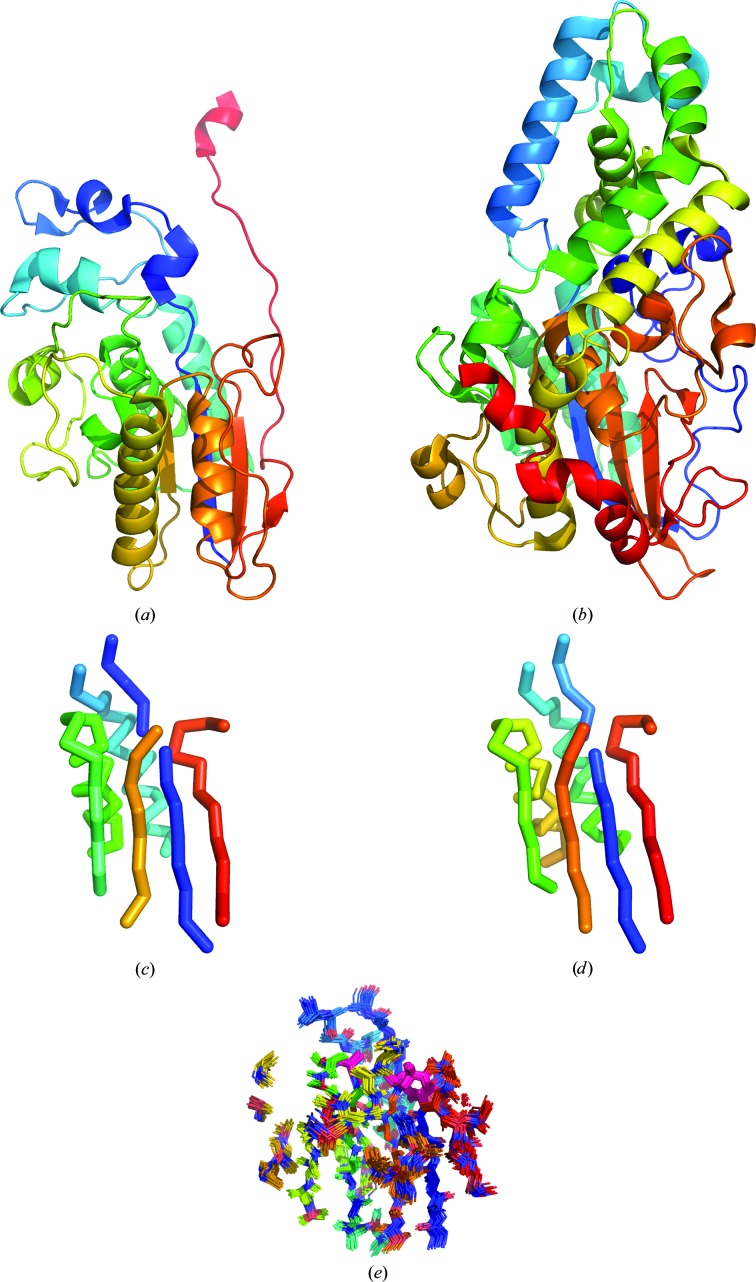
The structurally divergent homologues (*a*) ecdysone phosphate phosphatase (PDB entry 3c7t) and (*b*) phytase (PDB entry 1qwo), coloured as a spectrum from blue (N-terminus) to red (C-terminus), share a 73-residue core, as shown in (*c*) and (*d*), respectively, containing the characteristic catalytic His and Arg residues shown as sticks. A similar 90-residue ensemble polyalanine search model derived from *CONCOORD* processing of 3c7t (*e*) could solve the structure of 1qwo and contained a structural core containing the catalytic His and Arg positions (pink sticks).

Most remarkable of all was the solution in the same way of target 1qwo from branch 2 of the family (Fig. 1 ▸). The two branches are exceedingly distantly related (Rigden, 2008 ▸). For example, 1qwo and 3c7t share only 11% sequence identity, and less than a third of the target 1qwo can be structurally superimposed on 3c7t by *GESAMT* with a 1.85 Å C^α^ r.m.s.d. Success was obtained with a polyalanine ensemble search model of 90 residues that captures well the shared catalytic core between the two structures (Fig. 1 ▸). By residue, the search model contains only 35% of the originating structure 3c7t and 21% of the target. Since the search model was stripped back to polyalanine, the atomic comparison is even more striking: the search model contains 438 non-H atoms (CNOS), only 13% of the 3364 in the target. The sporadic success and unpredictable nature of the successful search models in the hardest cases, both in size and side-chain treatment, again illustrates the advantages of *AMPLE*’s automatic sampling of numerous variant search models.

### 3.3. Editing 3c7t using packing and rigidity metrics   

**Figure 2:**
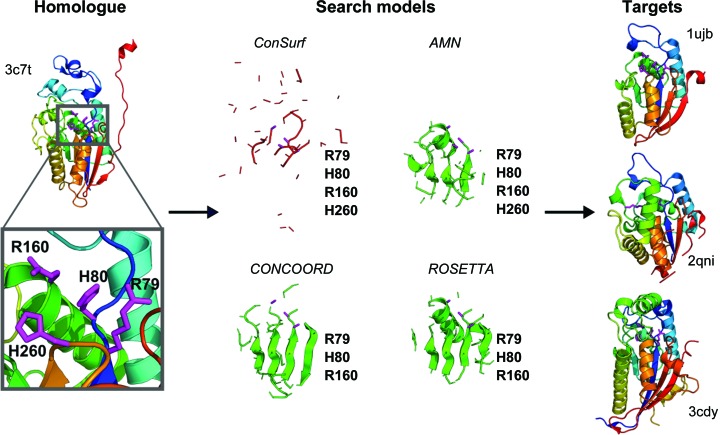
Overview of the single-homologue truncation mode of *AMPLE*. The distant homologue, in this case PDB entry 3c7t, is shown on the left coloured from blue (N-terminus) to red (C-terminus) with the catalytic His and Arg residues, which are conserved across the superfamily, coloured magenta, shown as sticks and labelled in the inset. A selection of truncations down to 15% of residues remaining, driven by the metrics shown, produces the search models shown in the centre. With the exception of *ConSurf*, the truncation produces well defined cores containing three or four catalytic residues, shown in magenta and labelled, that solve the distantly homologous targets shown on the right.

Given the success of the *CONCOORD*-derived ensemble search models, we wondered whether an even simpler approach, whereby metrics of packing and rigidity, acting as proxies for evolutionary conservation, could be used to edit the PDB structure 3c7t. Using the observed structural variance among the *CONCOORD* ensemble but applying it to truncate a single structure would also provide an interesting view on the value or otherwise of generating a search-model ensemble. As outlined in §[Sec sec2]2, we explored a variety of metrics, some requiring additional software, but others available as simple downloads from online resources (see Supplementary Table S2). For comparison, we also used the crystallographic *B* factors and solvent-accessible surface area (ASA; Bunkóczi & Read, 2011 ▸) of the deposited structure 3c7t. This procedure, depicted in Fig. 2 ▸, resulted in solution of four of the test cases (Table 3 ▸)

Overall, MR with edited single structures, rather than the ensembles employed above, is strikingly less successful (Table 3 ▸). We first explored some familiar scores previously used for this purpose (Bunkóczi & Read, 2011 ▸) and found them to perform very poorly. The search models from ASA-driven editing solved no structures. Using the crystallographic *B* factor as a guide, trimming first regions with high values solves only one member of the test set, 1ujb. Using sequence conservation directly, as obtained from *ConSurf*, is even worse, solving no structures. This might be owing to the fact that the conservation score gives a very jagged profile score, so that search models resulting from its use are very fragmentary (see, for example, Fig. 2 ▸). It is possible that a smoothed version of the score would perform better. Not previously explored, to our knowledge, sequence covariance is known to inform on packing and can be used for prediction of the functional sites (see, for example, Hopf *et al.*, 2012 ▸) that are likely to be better conserved between homologues. However, at least in the SMRF formulation (Jeong & Kim, 2016 ▸) used here, this approach did not produce successful search models.

Novel metrics based on rigidity or packing as a proxy for evolutionary conservation did better, consistently solving the same set of four targets: 1ujb, 2qni, 3dcy and 1qwo. However, there were differences in the ease of structure solution with regard to the number of successful search models in the set of 60 trialled throughout. For example, only a single *CABS-flex*-guided search model succeeded with 3dcy, whereas ten WCN-guided search models were successful. In practice, this is likely to correspond to a somewhat speedier structure solution, with a shorter time to first success, in the latter case. Remarkably, four metrics produced search models that could solve the very distant branch 2 homologue 1qwo, although fewer search models succeeded in general than solved the branch 1 targets (Table 3 ▸). For example, search models edited according to the weighted contact number (WCN) metric solved branch 1 targets 1ujb, 2qni and 3dcy 17, 22 and 10 times, respectively, but only two succeeded with 1qwo.

It is important to remember that manually edited versions of 3c7t, further processed in diverse ways with *MrBUMP*, only solved one of these structures: 2qni. Thus, the fact that a single structure can be automatically processed to search models over a range of sizes, using the novel metrics explored here, and readily solve two more represents a real advance. However, the *CONCOORD*-derived ensembles solved a further two, albeit only when a relatively large number of search models (540) were trialled (Table 2 ▸). This suggests a distinct advantage of computationally generated ensembles over edited single structures. *CONCOORD* is a computationally inexpensive method that takes 3 min on 16 cores for the 259-residue 3c7t, for example. Both it and *DSSP*, which it requires, are freely available so that crystallo­graphers may easily explore this possibility.

### 3.4. Additional examples   

The *CONCOORD* approach was tried on two difficult cases on which one of the authors (RK) collaborated, with the structures now deposited as PDB entries 4p9g and 5hxg. In each case runs of the automated pipelines *MrBUMP* (Keegan & Winn, 2008 ▸; Keegan *et al.*, 2018 ▸) and *BALBES* (Long *et al.*, 2008 ▸) both failed to solve the structures. The former structure, 4p9g, could eventually be solved with difficulty by an expert using conventional MR and a search model comprising a superposition of four edited homologous crystal structures. The latter could not be solved conventionally using available search models.

The structure of the jelly-roll fold enzyme 2,4′-dihydroxy­acetophenone dioxygenase from *Alcaligenes* sp. (PDB entry 4p9g; Keegan *et al.*, 2014 ▸) determined at 2.0 Å resolution was solved with a *CONCOORD*-produced ensemble derived from the closest available homologue: an uncharacterized protein from *Ralstonia eutropha* (PDB entry 3ebr; Joint Center for Structural Genomics, unpublished work) bearing only around 28% sequence identity to the target. In a full trial of all search models, around 60% were successful and contained between 29 and 157 residues. Failing search models contained between five and 157 residues. Both sets contained search models that derived from each of the three different modes of side-chain processing. The overall similarities between the two sets confirm that *AMPLE*’s automated sampling across a variety of variant search models is advantageous. When the same job was run to first solution, as would be more typical, the first success was achieved in 40 min on 14 processors of a workstation.

**Figure 3:**
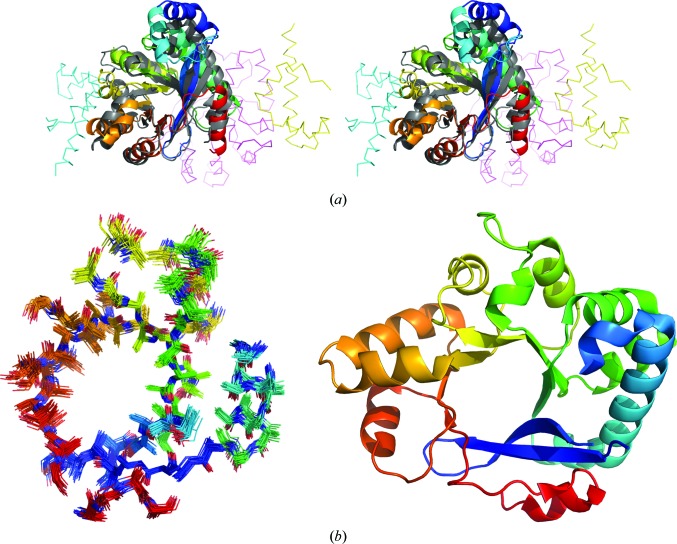
(*a*) Cross-eyed stereo comparison of the two EAL-domain proteins. 4hjf is shown as a cartoon coloured from blue to red from the N-terminus to the C-terminus. Chain *A* of the target, now deposited with PDB code 5hxg, is shown as a grey cartoon: the other chains in the two-heterodimer asymmetric unit are shown as differently coloured ribbons. (*b*) Comparison of one of the successful search models, an 81-residue ensemble, derived from the processing of a set of structures derived by processing 4hjf with *CONCOORD* (left) with, on the same scale, chain *A* of the solved target 5hxg (right), each coloured from blue to red from the N-terminus to the C-terminus.

The 2 Å resolution complex crystal structure now deposited with PDB code 5hxg was particularly challenging since it contained two copies of the heterodimer in the asymmetric unit. The uncharacterized EAL-domain protein contains 235 residues, while its interaction partner, a transcriptional regulator, contained 116 residues. It was solved using another EAL-domain protein with PDB code 4hjf (Midwest Center for Structural Genomics, unpublished work). This, the closest structurally characterized homologue, bore only 22% sequence identity to the target, and structure comparison post-solution shows that while the central β-barrel is relatively well conserved, differences in the length and orientation of loops and termini hampered routine structure solution (Fig. 3 ▸). In this relatively demanding case, not all search models were trialled. Success was achieved after about 36 h on 14 processors of a workstation using automatically derived ensembles containing 81 or 94 polyalanine residues which had captured the relatively structurally conserved β-barrel structure (Fig. 3 ▸). At that point 30 search models containing between 51 and 94 residues had been processed without leading to solution.

## 4. Conclusions   

It is relatively common to find that a new target bears only distant homology to its closest relative in the PDB. In these circumstances, conventional MR can be time-consuming, dependent on the availability of local expertise, and ultimately unsuccessful, and a wide variety of approaches have been tested to try to automatically extend the range of proteins that can be solved successfully (Keegan & Winn, 2007 ▸, 2008 ▸; Keegan *et al.*, 2011 ▸; Long *et al.*, 2008 ▸; Bunkóczi *et al.*, 2013 ▸; Vagin & Lebedev, 2015 ▸). The work presented here addresses this situation in two ways, based on the recently elucidated correlation between protein structural packing and flexibility and on local rates of evolutionary conservation (Shih *et al.*, 2012 ▸; Yeh *et al.*, 2014 ▸). Firstly, we explore the building of an ensemble of structures based on the distant homologue, using distance geometry methods in *CONCOORD*. To our knowledge, this is the first such approach whereby a single structure is meaningfully transformed into an ensemble for the purposes of MR, although normal-mode prediction as a means for conformational sampling has a long history of application in MR (McCoy *et al.*, 2013 ▸; Suhre & Sanejouand, 2004*a* ▸,*b* ▸). Processing of these *CONCOORD*-derived structures by *AMPLE* in the same way that *ab initio* models are dealt with produces ensemble search models that can solve some exceedingly distant homology cases. Alternative algorithms at each of the two steps involved (ensemble generation and per-residue measurement of structural diversity) could be explored in the future. Secondly, we present a new *AMPLE* single-structure mode that provides an automated way to sample multiple knowledge-based derivatives of a single distant homologue. The sampling is driven by a per-residue score file, obtained by the user (Supplementary Table S2), which for best performance contains figures reflecting packing or predicted flexibility along the chain. Although performance is less good than with the *CONCOORD*-derived ensembles, we showed that this can solve targets that were intractable with expertly manually derived structures further edited in various ways by *MrBUMP*. Several avenues exist for further development. For example, it may be possible to use target–homologue alignments to enable a more sophisticated treatment of side-chain editing. Providing a variable r.m.s.d. error estimate to *Phaser* that depends on the degree of truncation applied to produce the search model is also worth exploring. Finally, we note that new metrics that may offer improved performance in single-structure editing mode continue to be developed (Liu *et al.*, 2017 ▸).

## Supplementary Material

Supplementary Figures and Tables.. DOI: 10.1107/S2059798318002310/di5015sup1.pdf

## Figures and Tables

**Table 1:** Characteristics of the test set of proteins

Branch of the His phosphatase superfamily	PDB code	Length in crystal structure	Resolution (Å)	Solvent content (%)	Name	*GESAMT* *versus* 3c7t, *Q*-score†	*TM-align* alignment with 3c7t, TM-score†
1	3c7t	259	1.8	49	*Bombyx mori* ecdysone phosphate phosphatase	—	—
	1ujb	156	2.1	44	*Escherichia coli* SixA	0.35 (139, 1.85, 20.9)	0.51 (151, 2.63, 21.2)
	2qni	194	1.8	63	Uncharacterized *Agrobacterium fabrum* protein Atu02999	0.30 (148, 2.08, 13.5)	0.53 (162, 2.94, 12.3)
	1e59	239	1.3	51	*Escherichia coli* phosphoglycerate mutase	0.30 (174, 2.37, 17.8)	0.60 (190, 3.51, 17.9)
	4eo9	240	2.45	62	*Mycobacterium leprae* phosphoglycerate mutase	0.30 (173, 2.34, 18.5)	0.61 (190, 3.48, 17.4)
	1ebb	202	2.3	54	*Bacillus stearothermophilus* PhoE	0.31 (156, 2.18, 21.2)	0.56 (168, 2.94, 21.4)
	3dcy	269	1.75	41	Human TIGAR	0.24 (150, 2.20, 20.7)	0.54 (168, 3.44, 19.6)
2	1qwo	434	1.5	48	*Aspergillus fumigatus* phytase	0.10 (153, 3.05, 14.4)	0.51 (176, 4.48, 11.9)
	1dkm	410	2.25	53	*Escherichia coli* phytase	0.12 (151, 2.71, 17.2)	0.51 (177, 4.30, 14.7)

† The values in parentheses are the length matched, the r.m.s.d. in Å and the percentage sequence identity in the matched region.

**Table 2:** Solution of distantly homologous targets using search models derived from *CONCOORD* ensembles

Branch of the His phosphatase superfamily	PDB code	Overall sequence identity to 3c7t in *MAFFT *sequence alignment (%)	*GESAMT* structural alignment: No. of superimposed residues, C^α^ r.m.s.d.	Length in crystal structure	Resolution (Å)	No. of successful search models†	Residues in successful search models (No. of solutions with that number of residues)	Side-chain treatments in successful search models (No. of solutions for each side-chain treatment)	*SHELXE* CC¶	*SHELXE* ACL¶	Final *R*_free_ ¶	Solved by *MrBUMP* with the 3c7t crystal structure or manually derived derivatives?
1	1ujb	24	139, 1.85	156	2.1	14/180	51 (7), 64 (2), 77 (3), 103 (2)	PolyAla (10), reliable (3), all atom (1)	48.66–52.28	36–155	0.235–0.259	No
	2qni	12	148, 2.08	194	1.8	61/180	25 (9), 38 (9), 51 (9), 64 (9), 77 (6), 90 (6), 103 (6), 116 (3), 129 (3), 142 (1)	PolyAla (27), reliable (21), all atom (13)	45.89–49.21	29–62	0.215–0.320	Yes
	1e59	17	174, 2.37	239	1.3	0/540	—	—	—	—	—	No
	4eo9	18	173, 2.34	240	2.45	1/540	90	Reliable	39.45	38	0.230	No
		19	156, 2.18	202	2.3	1/540	77	PolyAla	24.72	16	0.232††	No
	3dcy	18	150, 2.20	269	1.75	5/180	38, 51 (3), 64	PolyAla (5)	46.13–46.99	37–79	0.302–0.317	No
2	1qwo	11	153, 3.05	434	1.5	1/540	90	PolyAla	43.61	83	0.216	No
	1dkm	16	151, 2.71	410	2.25	0/540	—	—	—	—	—	No

† A denominator of 180 means that success was achieved among the 180 search models deriving from the first cluster of *CONCOORD* outputs for 3c7t. A denominator of 540 means that the first cluster did not produce a successful search model and that search models from clusters 2 and 3 were therefore trialled.

‡ The range is shown for multiple solutions.

§ Required additional rounds of *ARP*/*wARP* or *Buccaneer* to obtain this value.

**Table 3:** Solution of distantly homologous targets using an edited version of the single structure of 3c7t The editing was driven by a variety of metrics relating to per-residue packing, rigidity, sequence conservation and *B* factors (see Supplementary Table S2 for details). The number of successful search models resulting from 20 truncation levels and three side-chain treatments is shown. A dash indicates failure to solve a target.

				Metrics driving editing of a single structure of 3c7t for MR in *AMPLE*											
				Structure-derived predicted properties				Sequence-derived properties				Crystallographic properties			
				Rigidity				Packing		Others			Sequence conservation	Sequence covariance	
Branch of the His phosphatase superfamily	PDBcode	Solved by *MRBump* with manual edits?	Solved with *CONCOORD* ensembles?	*AMN*	*CABS-flex*	*CONCOORD*	*WEBnm@*	*ROSETTA*	WCN†	Relative solvent accessibility	*ResQ *predicted *B* factor (Å^2^)	*ResQ* predicted structural quality	*ConSurf*	SMRF (contacts)	Crystallographic *B* factor
1	1ujb	No	Yes	12	6	10	8	23	17	—	11	4	—	—	6
	2qni	Yes	Yes	19	14	18	23	17	22	—	15	8	—	—	—
	1e59	No	No	—	—	—	—	—	—	—	—	—	—	—	—
	4e09	No	Yes	—	—	—	—	—	—	—	—	—	—	—	—
		No	Yes	—	—	—	—	—	—	—	—	—	—	—	—
	3dcy	No	Yes	3	1	7	6	6	10	—	1	5	—	—	—
2	1qwo	No	Yes	—	5	2	—	4	2	—	—	—	—	—	—
	1dkm	No	No	—	—	—	—	—	—	—	—	—	—	—	—

† This metric was also applied to a structure with *B* factors uniformly set to 20 with little effect on success.

## References

[bb1] Abergel, C. (2013). *Acta Cryst.* D**69**, 2167–2173. 10.1107/S0907444913015291 PMC3817689 24189227

[bb2] Adams, P. D. *et al.* (2010). *Acta Cryst.* D**66**, 213–221.

[bb3] Altschul, S. F., Madden, T. L., Schäffer, A. A., Zhang, J., Zhang, Z., Miller, W. & Lipman, D. J. (1997). *Nucleic Acids Res.* **25**, 3389–3402. 10.1093/nar/25.17.3389 PMC146917 9254694

[bb4] Angermüller, C., Biegert, A. & Söding, J. (2012). *Bioinformatics*, **28**, 3240–3247. 10.1093/bioinformatics/bts622 23080114

[bb5] Ashkenazy, H., Abadi, S., Martz, E., Chay, O., Mayrose, I., Pupko, T. & Ben-Tal, N. (2016). *Nucleic Acids Res.* **44**, W344–W350. 10.1093/nar/gkw408 PMC4987940 27166375

[bb99] Baugh, L. *et al.* (2015). *Tuberculosis*, **95**, 142–148.

[bb6] Bibby, J., Keegan, R. M., Mayans, O., Winn, M. D. & Rigden, D. J. (2012). *Acta Cryst.* D**68**, 1622–1631. 10.1107/S0907444912039194 23151627

[bb7] Bibby, J., Keegan, R. M., Mayans, O., Winn, M. D. & Rigden, D. J. (2013). *Acta Cryst.* D**69**, 2194–2201. 10.1107/S0907444913018453 PMC3817692 24189230

[bb8] Bunkóczi, G., Echols, N., McCoy, A. J., Oeffner, R. D., Adams, P. D. & Read, R. J. (2013). *Acta Cryst.* D**69**, 2276–2286. 10.1107/S0907444913022750 PMC3817702 24189240

[bb9] Bunkóczi, G. & Read, R. J. (2011). *Acta Cryst.* D**67**, 303–312. 10.1107/S0907444910051218 PMC3069745 21460448

[bb75] Chen, Y., Jakoncic, J., Wang, J., Zheng, X., Carpino, N. & Nassar, N. (2008). *Biochemistry*, **47**, 12135–12145. 10.1021/bi801318w PMC2722925 18937503

[bb10] Cowtan, K. (2006). *Acta Cryst.* D**62**, 1002–1011. 10.1107/S0907444906022116 16929101

[bb11] Eyal, E., Lum, G. & Bahar, I. (2015). *Bioinformatics*, **31**, 1487–1489. 10.1093/bioinformatics/btu847 PMC4410662 25568280

[bb12] Finn, R. D., Coggill, P., Eberhardt, R. Y., Eddy, S. R., Mistry, J., Mitchell, A. L., Potter, S. C., Punta, M., Qureshi, M., Sangrador-Vegas, A., Salazar, G. A., Tate, J. & Bateman, A. (2016). *Nucleic Acids Res.* **44**, D279–D285. 10.1093/nar/gkv1344 PMC4702930 26673716

[bb13] Fu, L., Niu, B., Zhu, Z., Wu, S. & Li, W. (2012). *Bioinformatics*, **28**, 3150–3152. 10.1093/bioinformatics/bts565 PMC3516142 23060610

[bb14] Groot, B. L. de, van Aalten, D. M. F., Scheek, R. M., Amadei, A., Vriend, G. & Berendsen, H. J. C. (1997). *Proteins*, **29**, 240–251. 10.1002/(sici)1097-0134(199710)29:2<240::aid-prot11>3.0.co;2-o 9329088

[bb15] Hopf, T. A., Colwell, L. J., Sheridan, R., Rost, B., Sander, C. & Marks, D. S. (2012). *Cell*, **149**, 1607–1621. 10.1016/j.cell.2012.04.012 PMC3641781 22579045

[bb16] Huang, T.-T., Hwang, J.-K., Chen, C.-H., Chu, C.-S., Lee, C.-W. & Chen, C.-C. (2015). *Nucleic Acids Res.* **43**, W338–W342. 10.1093/nar/gkv454 PMC4489310 25943546

[bb17] Jamroz, M., Kolinski, A. & Kmiecik, S. (2013). *Nucleic Acids Res.* **41**, W427–W431. 10.1093/nar/gkt332 PMC3692091 23658222

[bb18] Jaroszewski, L., Rychlewski, L., Li, Z., Li, W. & Godzik, A. (2005). *Nucleic Acids Res.* **33**, W284–W288. 10.1093/nar/gki418 PMC1160179 15980471

[bb19] Jeong, C.-S. & Kim, D. (2016). *BMC Bioinformatics*, **17**, 99. 10.1186/s12859-016-0948-2 PMC4765150 26911566

[bb20] Jorgensen, W. L., Maxwell, D. S. & Tirado-Rives, J. (1996). *J. Am. Chem. Soc.* **118**, 11225–11236.

[bb21] Kabsch, W. & Sander, C. (1983). *Biopolymers*, **22**, 2577–2637. 10.1002/bip.360221211 6667333

[bb22] Katoh, K. & Standley, D. M. (2013). *Mol. Biol. Evol.* **30**, 772–780. 10.1093/molbev/mst010 PMC3603318 23329690

[bb23] Keegan, R. M., Bibby, J., Thomas, J., Xu, D., Zhang, Y., Mayans, O., Winn, M. D. & Rigden, D. J. (2015). *Acta Cryst.* D**71**, 338–343. 10.1107/S1399004714025784 PMC4321487 25664744

[bb24] Keegan, R., Lebedev, A., Erskine, P., Guo, J., Wood, S. P., Hopper, D. J., Rigby, S. E. J. & Cooper, J. B. (2014). *Acta Cryst.* D**70**, 2444–2454. 10.1107/S1399004714015053 PMC4219425 25195757

[bb25] Keegan, R. M., Long, F., Fazio, V. J., Winn, M. D., Murshudov, G. N. & Vagin, A. A. (2011). *Acta Cryst.* D**67**, 313–323. 10.1107/S0907444911007530 PMC3069746 21460449

[bb26] Keegan, R. M., McNicholas, S. J., Thomas, J. M. H., Simpkin, A. J., Simkovic, F., Uski, V., Ballard, C. C, Winn, M. D., Wilson, K. S. & Rigden, D. J. (2018). *Acta Cryst.* D**74**, 167–182. 10.1107/S2059798318003455 PMC5947758 29533225

[bb27] Keegan, R. M. & Winn, M. D. (2007). *Acta Cryst.* D**63**, 447–457. 10.1107/S0907444907002661 17372348

[bb28] Keegan, R. M. & Winn, M. D. (2008). *Acta Cryst.* D**64**, 119–124. 10.1107/S0907444907037195 PMC2394800 18094475

[bb29] Krissinel, E. (2012). *J. Mol. Biochem.* **1**, 76–85. PMC5117261 27882309

[bb30] Krissinel, E. & Henrick, K. (2007). *J. Mol. Biol.* **372**, 774–797. 10.1016/j.jmb.2007.05.022 17681537

[bb31] Krivov, G. G., Shapovalov, M. V. & Dunbrack, R. L. Jr (2009). *Proteins*, **77**, 778–795. 10.1002/prot.22488 PMC2885146 19603484

[bb32] Langer, G., Cohen, S. X., Lamzin, V. S. & Perrakis, A. (2008). *Nature Protoc.* **3**, 1171–1179. 10.1038/nprot.2008.91 PMC2582149 18600222

[bb33] Leaver-Fay, A. *et al.* (2011). *Methods Enzymol.* **487**, 545–574. 10.1016/B978-0-12-381270-4.00019-6 PMC4083816 21187238

[bb34] Lebedev, A. A., Vagin, A. A. & Murshudov, G. N. (2008). *Acta Cryst.* D**64**, 33–39. 10.1107/S0907444907049839 PMC2394799 18094465

[bb35] Liu, J.-W., Cheng, C.-W., Lin, Y.-F., Chen, S.-Y., Hwang, J.-K. & Yen, S.-C. (2017). *Biochim. Biophys. Acta*, **1866**, 379–386. 10.1016/j.bbapap.2017.09.003 28911812

[bb36] Long, F., Vagin, A. A., Young, P. & Murshudov, G. N. (2008). *Acta Cryst.* D**64**, 125–132. 10.1107/S0907444907050172 PMC2394813 18094476

[bb37] McCoy, A. J. (2004). *Acta Cryst.* D**60**, 2169–2183. 10.1107/S0907444904016038 15572770

[bb38] McCoy, A. J., Grosse-Kunstleve, R. W., Adams, P. D., Winn, M. D., Storoni, L. C. & Read, R. J. (2007). *J. Appl. Cryst.* **40**, 658–674. 10.1107/S0021889807021206 PMC2483472 19461840

[bb39] McCoy, A. J., Grosse-Kunstleve, R. W., Storoni, L. C. & Read, R. J. (2005). *Acta Cryst.* D**61**, 458–464. 10.1107/S0907444905001617 15805601

[bb40] McCoy, A. J., Nicholls, R. A. & Schneider, T. R. (2013). *Acta Cryst.* D**69**, 2216–2225. 10.1107/S0907444913021811 PMC3817695 24189233

[bb41] McCoy, A. J., Oeffner, R. D., Wrobel, A. G., Ojala, J. R., Tryggvason, K., Lohkamp, B. & Read, R. J. (2017). *Proc. Natl Acad. Sci. USA*, **114**, 3637–3641. 10.1073/pnas.1701640114 PMC5389281 28325875

[bb42] McNicholas, S., Potterton, E., Wilson, K. S. & Noble, M. E. M. (2011). *Acta Cryst.* D**67**, 386–394. 10.1107/S0907444911007281 PMC3069754 21460457

[bb43] Oeffner, R. D., Bunkóczi, G., McCoy, A. J. & Read, R. J. (2013). *Acta Cryst.* D**69**, 2209–2215. 10.1107/S0907444913023512 PMC3817694 24189232

[bb44] Park, H., DiMaio, F. & Baker, D. (2015). *Structure*, **23**, 1123–1128. 10.1016/j.str.2015.03.022 PMC4456269 25960407

[bb45] Read, R. J. & McCoy, A. J. (2016). *Acta Cryst.* D**72**, 375–387. 10.1107/S2059798315013236 PMC4784668 26960124

[bb46] Rigden, D. J. (2008). *Biochem. J.* **409**, 333–348. 10.1042/BJ20071097 18092946

[bb47] Rodríguez, D., Sammito, M., Meindl, K., de Ilarduya, I. M., Potratz, M., Sheldrick, G. M. & Usón, I. (2012). *Acta Cryst.* D**68**, 336–343. 10.1107/S0907444911056071 PMC3322593 22505254

[bb48] Rossmann, M. G. & Blow, D. M. (1962). *Acta Cryst.* **15**, 24–31.

[bb49] Sammito, M., Meindl, K., de Ilarduya, I. M., Millán, C., Artola-Recolons, C., Hermoso, J. A. & Usón, I. (2014). *FEBS J.* **281**, 4029–4045. 10.1111/febs.12897 24976038

[bb50] Sammito, M., Millán, C., Rodríguez, D. D., de Ilarduya, I. M., Meindl, K., De Marino, I., Petrillo, G., Buey, R. M., de Pereda, J. M., Zeth, K., Sheldrick, G. M. & Usón, I. (2013). *Nature Methods*, **10**, 1099–1101. 10.1038/nmeth.2644 24037245

[bb51] Schäffer, A. A., Aravind, L., Madden, T. L., Shavirin, S., Spouge, J. L., Wolf, Y. I., Koonin, E. V. & Altschul, S. F. (2001). *Nucleic Acids Res.* **29**, 2994–3005. 10.1093/nar/29.14.2994 PMC55814 11452024

[bb52] Schwarzenbacher, R., Godzik, A., Grzechnik, S. K. & Jaroszewski, L. (2004). *Acta Cryst.* D**60**, 1229–1236. 10.1107/S0907444904010145 15213384

[bb53] Shih, C.-H., Chang, C.-M., Lin, Y.-S., Lo, W.-C. & Hwang, J.-K. (2012). *Proteins*, **80**, 1647–1657. 10.1002/prot.24058 22454236

[bb54] Shortle, D., Simons, K. T. & Baker, D. (1998). *Proc. Natl Acad. Sci. USA*, **95**, 11158–11162. 10.1073/pnas.95.19.11158 PMC21612 9736706

[bb55] Simkovic, F., Thomas, J. M. H., Keegan, R. M., Winn, M. D., Mayans, O. & Rigden, D. J. (2016). *IUCrJ*, **3**, 259–270. 10.1107/S2052252516008113 PMC4937781 27437113

[bb56] Stein, N. (2008). *J. Appl. Cryst.* **41**, 641–643.

[bb57] Storoni, L. C., McCoy, A. J. & Read, R. J. (2004). *Acta Cryst.* D**60**, 432–438. 10.1107/S0907444903028956 14993666

[bb58] Suhre, K. & Sanejouand, Y.-H. (2004*a*). *Nucleic Acids Res.* **32**, W610–W614. 10.1093/nar/gkh368 PMC441506 15215461

[bb59] Suhre, K. & Sanejouand, Y.-H. (2004*b*). *Acta Cryst.* D**60**, 796–799. 10.1107/S0907444904001982 15039589

[bb60] Terwilliger, T. C., DiMaio, F., Read, R. J., Baker, D., Bunkóczi, G., Adams, P. D., Grosse-Kunstleve, R. W., Afonine, P. V. & Echols, N. (2012). *J. Struct. Funct. Genomics*, **13**, 81–90. 10.1007/s10969-012-9129-3 PMC3375004 22418934

[bb61] Terwilliger, T. C., Grosse-Kunstleve, R. W., Afonine, P. V., Moriarty, N. W., Zwart, P. H., Hung, L.-W., Read, R. J. & Adams, P. D. (2008). *Acta Cryst.* D**64**, 61–69. 10.1107/S090744490705024X PMC2394820 18094468

[bb62] Theobald, D. L. & Wuttke, D. S. (2006). *Bioinformatics*, **22**, 2171–2172. 10.1093/bioinformatics/btl332 PMC2584349 16777907

[bb63] Thorn, A. & Sheldrick, G. M. (2013). *Acta Cryst.* D**69**, 2251–2256. 10.1107/S0907444913027534 PMC3817699 24189237

[bb64] Tiwari, S. P., Fuglebakk, E., Hollup, S. M., Skjaerven, L., Cragnolini, T., Grindhaug, S. H., Tekle, K. M. & Reuter, N. (2014). *BMC Bioinformatics*, **15**, 427. 10.1186/s12859-014-0427-6 PMC4339738 25547242

[bb65] Vagin, A. & Lebedev, A. (2015). *Acta Cryst.* A**71**, s19.

[bb66] Vagin, A. & Teplyakov, A. (2010). *Acta Cryst.* D**66**, 22–25. 10.1107/S0907444909042589 20057045

[bb67] Winn, M. D. *et al.* (2011). *Acta Cryst.* D**67**, 235–242.

[bb68] Yang, J., Wang, Y. & Zhang, Y. (2016). *J. Mol. Biol.* **428**, 693–701. 10.1016/j.jmb.2015.09.024 PMC4783266 26437129

[bb69] Yeh, S.-W., Liu, J.-W., Yu, S.-H., Shih, C.-H., Hwang, J.-K. & Echave, J. (2014). *Mol. Biol. Evol.* **31**, 135–139. 10.1093/molbev/mst178 24109601

[bb70] Zhang, Y. & Skolnick, J. (2004). *J. Comput. Chem.* **25**, 865–871. 10.1002/jcc.20011 15011258

[bb71] Zhang, Y. & Skolnick, J. (2005). *Nucleic Acids Res.* **33**, 2302–2309. 10.1093/nar/gki524 PMC1084323 15849316

