# Supplementary material for: Ensembles generated from crystal structures of single distant homologues solve challenging molecular-replacement cases in AMPLE
Source: Acta Crystallogr D Struct Biol. 2018 Mar 2;74(Pt 3):183–93. doi: 10.1107/S2059798318002310 (PMC5947759; doi:10.1107/S2059798318002310)
Supplement: Supplementary file 1 [file d-74-00183-sup1.pdf]

STRUCTURAL BIOLOGY

Volume 74 (2018)

Supporting information for article:

Ensembles generated from crystal structures of single distant homologues solve challenging molecular-replacement cases in *AMPLE*

Daniel J. Rigden, Jens M. H. Thomas, Felix Simkovic, Adam Simpkin, Martyn D. Winn, Olga Mayans and Ronan M. Keegan

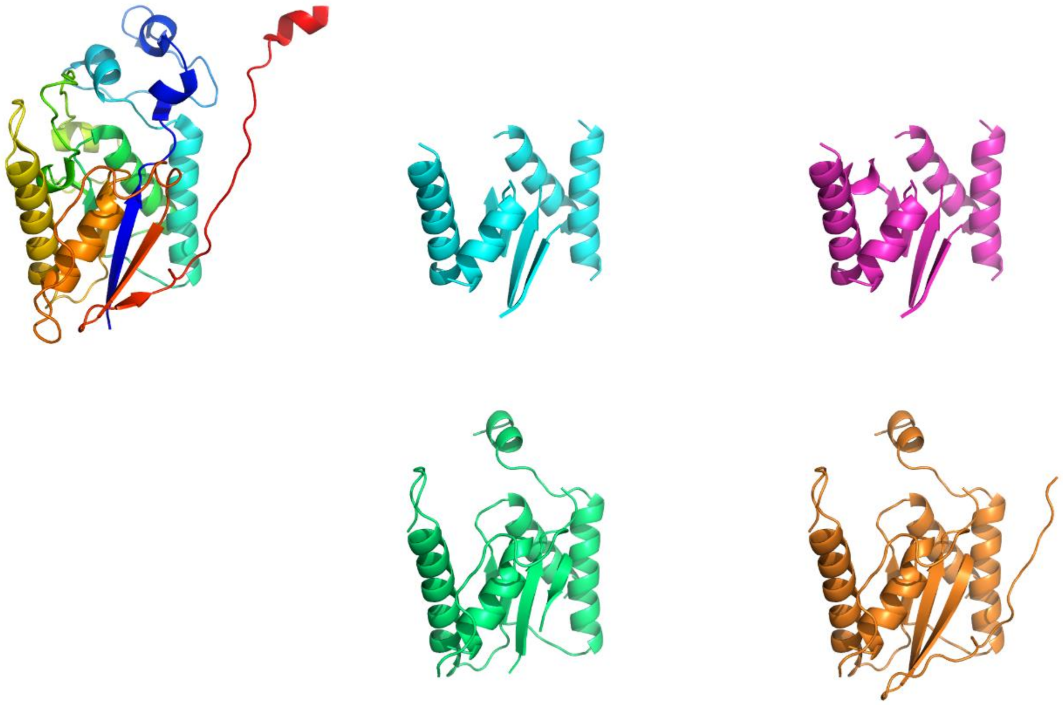

Figure S1 Comparison of chain A of 3c7t structure (left) with four manually edited versions trialled as search models (centre and right) containing 88 (cyan), 95 (magenta), 138 (green) and 159 (orange) residues.

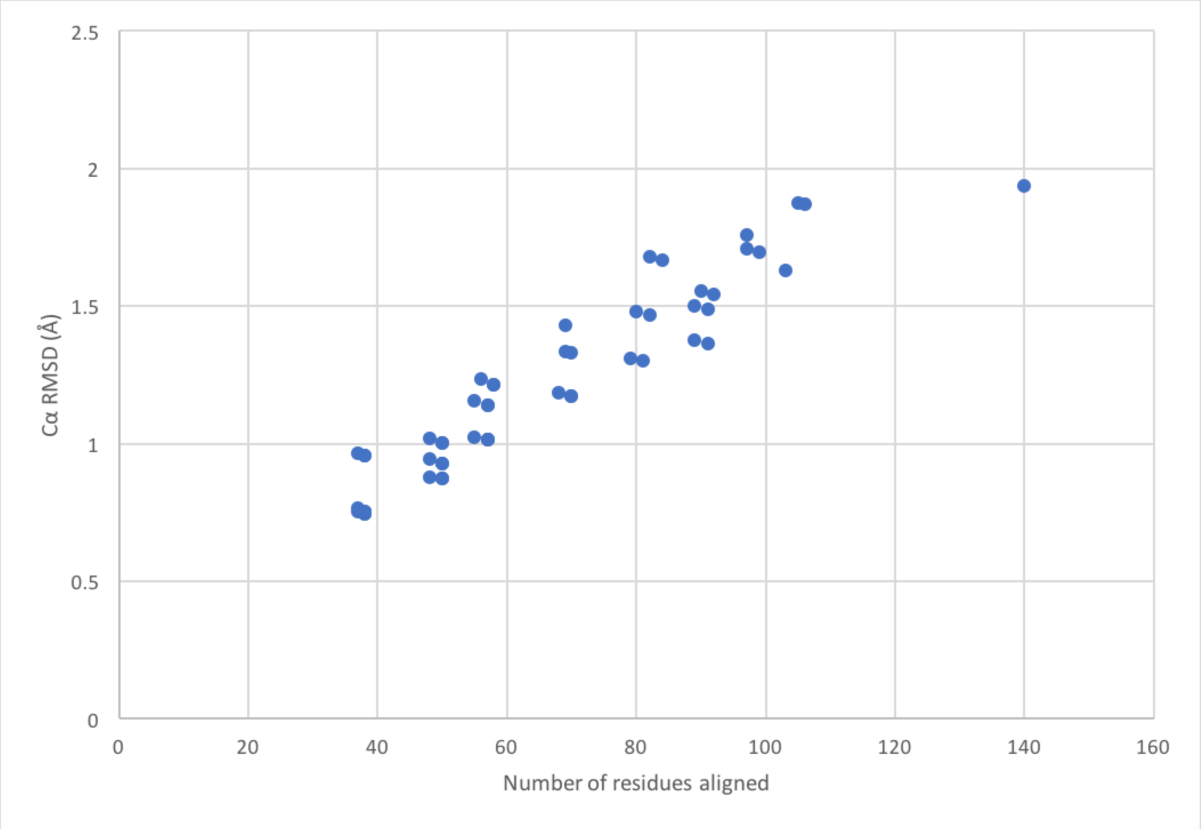

Figure S2 GESAMT superpositions of search model ensembles, each derived from 3c7t CONCOORD results and represented by its first member, that successfully solved target 2qni. Each superposition is shown according to number of residues aligned and rms deviation on aligned Cα atoms. The smallest, 25-residues search model ensembles could not be aligned to 3c7t with GESAMT.

Table S1 Structure comparisons between the four manually edited 3c7t derivatives at the targets whose solution was attempted.

For each an overall Q-score (on a range of 0-1) is shown and, in brackets, the number of residues aligned, their Cα rmsd and the % sequence identity of the alignment. Edited derivatives that led to successful MR, after side chain editing in MrBUMP, are shown in red.

Manually edited derivative of 3c7t	Number of residues in manually edited derivative of 3c7t (see Figure 1)	1e59	1ebb	1ujb	2qni	3dcy	4eo9	1dkm	1qwo
1	159	0.330 (138, 2.162, 0.188)	0.343 (128, 2.095, 0.234)	0.422 (119, 1.782, 0.227)	0.345 (123, 2.020, 0.146)	0.264 (123, 2.154, 0.203)	0.323 (135, 2.073, 0.200)	0.136 (123, 2.530, 0.179)	0.126 (130, 2.912, 0.123)
2	138	0.309 (123, 2.088, 0.211)	0.323 (113, 1.939, 0.257)	0.358 (107, 2.086, 0.262)	0.356 (110, 1.629, 0.155)	0.244 (109, 2.085, 0.229)	0.305 (122, 2.068, 0.221)	0.123 (106, 2.364, 0.198)	0.125 (120, 2.879, 0.133)
3	95	0.286 (94, 1.798, 0.181)	0.304 (88, 1.719, 0.227)	0.367 (90, 2.097, 0.244)	0.313 (85, 1.587, 0.141)	0.244 (89, 1.978, 0.180)	0.283 (94, 1.827, 0.202)	0.138 (89, 2.060, 0.169)	0.128 (91, 2.274, 0.154)
4	88	0.262 (87, 1.835, 0.184)	0.274 (81, 1.763, 0.222)	0.334 (81, 1.966, 0.222)	0.292 (79, 1.584, 0.139)	0.221 (82, 2.019, 0.183)	0.260 (87, 1.847, 0.207)	0.131 (83, 2.040, 0.205)	0.117 (84, 2.276, 0.143)

Table S2 Computational analyses used to drive single structure editing of 3c7t

Name of method in Table 3	Server or local analysis	URL of server or software	Biophysical basis of the metric	How to retrieve the pre-residue values
AMN	Server	http://anm.csb.pitt.edu/	Anisotropic Network Model predicts per-residue flexibility value.	Submit a PDB code or PDB file. Results include a file `oanm.bfactors` in which the fourth column are predicted B-factors. High predicted B-factors result from high predicted flexibility.
CABS-flex	Server	http://biocomp.chem.uw.edu.pl/CABSflex/	Coarse-grained protein structural fluctuation server	Submit a PDB code or PDB file. Results include a file `trajectory_fluct.txt` in which the second column contains per-residue flexibility predictions
CONCOORD	Local analysis	http://www3.mpibpc.mpg.de/groups/de_groot/concoord/	The software calculates conformational ensembles abiding by restraints derived from the input structure.	Structurally align the resulting structures with THESEUS (http://www.theseus3d.org/). In the resulting file `theseus_variances.txt`, the 5th column contain per-residue structural variance values. High values reflected poorly packed residues.
WEBnm@	Server	http://apps.cbu.uib.no/webnma	Normal Mode analysis predicts per-residue flexibility value.	Submit a PDB code or PDB file. Results include a file called, for example, `3c7tA_fluctuationsplot.dat` in which the second column contains per-residue fluctuation values.
Rosetta_fast	Local analysis	https://www.rosettacommons.org/	Regions which vary after a brief conformational sampling/refinement protocol will be less well packed	Sample command line would be<br>`relax.linuxgccrelease -database /path/to/database -in:file:s 3c7tA.pdb -in:file:fullatom -out:pdb -relax:fast -nstruct 100`

				Structurally align the resulting structures with THESEUS (http://www.theseus3d.org/). In the resulting file `theseus_variances.txt`, the 5th column contain per-residue structural variance values. High values reflected poorly packed residues.
WCN	Server	http://ps2v3.life.nctu.edu.tw/	Weighted Contact Number is a direct measure of packing.	Submit a PDB ID or structure and its sequence. In the results page download the WCN profile from the link. Its 3rd column contains rWCN (reciprocal WCN) values per residue suitable for input to AMPLE.
Relative solvent accessibility	Server	http://www.ebi.ac.uk/pdbe/pisa/	Exposed, solvent-accessible regions are likely to be more flexible.	Submit a PDB ID or coordinate file to the PISA server and choose Analyse. On results page choose Monomers and then click on Details foir the chain of interest. The 4th column in the resulting Table contains Accessible Surface Area in Å^2 suitable for AMPLE.
ResQ predicted B-factor	Server	https://zhanglab.ccmb.med.umich.edu/ResQ/	Designed to predict protein structure model quality, here applied for a different purpose. Directly measures structural variance between the submitted structure and similar structures discovered in the PDB, returning per-residue measures of predicted error and B-factors. High values of either indicate positions of high variability between	Submit a PDB file. In the results tarball the file local.txt contains a Table. Columns 6 and 9 contain predicted B-factors (column head rBF) and structural quality measurements (column head RSQ_3) suitable for AMPLE.
ResQ predicted structural quality	Server	https://zhanglab.ccmb.med.umich.edu/ResQ/		

			the set of similar structures collected.	
Consurf	Server	http://consurf.tau.ac.il/2016/	Directly assesses evolutionary conservation on a per-residue level by sequence analysis of an alignment of collected homologous sequences.	Follow the prompts and supply a PDB or structure. Follow further prompts and Submit. Eventual results contain a file called `consurf.grades` in which the 4th column contains evolutionary conservation (low values indicating high conservation) suitable for AMPLE.
SMRF (contacts)	Local analysis	https://github.com/jeongchans/smrf	Positional coevolution score to identify functional sites in protein structure through structure-based Markov Random Field. Functional sites are likely to be more structurally conserved than the remainder of the protein	Create a Multiple Sequence Alignment with HHblits from the HH-suite package (https://github.com/soedinglab/hh-suite), and provide this alignment alongside the target structure to the smrf.py script.
Crystallographic B-factor	Server	http://swift.cmbi.ru.nl/servers/html/index.html	Poorly packed and/or flexible regions are often evident by an elevated B-factor.	Choose Other options then Average B factors. Enter a PDB ID or provide a PDB file. In the Results average backbone B-factors per residue are provided.
